# Is It Time for an Expanded Role of Dexmedetomidine in Contemporary Anesthesia Practice? - A Clinician’s Perspective

**Published:** 2018-04-12

**Authors:** Christian Bohringer, Hong Liu

**Affiliations:** Department of Anesthesiology and Pain Medicine, University of California Davis Health, Sacramento, California, USA

**Keywords:** Dexmedetomidine, Anesthesia practice, Analgesia

## Abstract

Since its approval by US Food and Drug Administration in 1999 the clinical use of dexmedetomidine has been gaining in popularity. The indications and clinical applications of this drug have been expanded significantly. In this paper we reviewed its pharmacokinetics, pharmacodynamics, mechanisms of action and mainly focused on its clinical uses and outcomes.

Common clinical uses of dexmedetomidine include pre-operative anxiolysis, heart rate control during intubation, treatment of bronchospasm, prevention of laryngospasm and avoiding opioid-induced post-operative respiratory depression and nausea and vomiting. Avoiding opioid induced respiratory depression has been especially beneficial in patients with sleep apnea syndrome. Other problems that can be prevented with dexmedetomidine are tachydysrhythmias, myocardial ischemia, delirium and acute kidney injury. Dexmedetomidine is an excellent sedative drug for intubated patients and it greatly facilitates neurological evaluation. It has been used successfully as a patient-controlled anesthesia drug and to prevent shivering. It is also used as an adjuvant to local anesthetics. It has been suggested that dexmedetomidine is a drug that has many beneficial effects and should be used more frequently by anesthesia care providers to prevent common problems in the peri-operative period. With judicious titration to effect during the intravenous administration of this drug the occurrence of side effects can be minimized. It is very likely that this drug will ascend to take a much more prominent role in future anesthesia practice.

## Introduction

**Figure 1:**
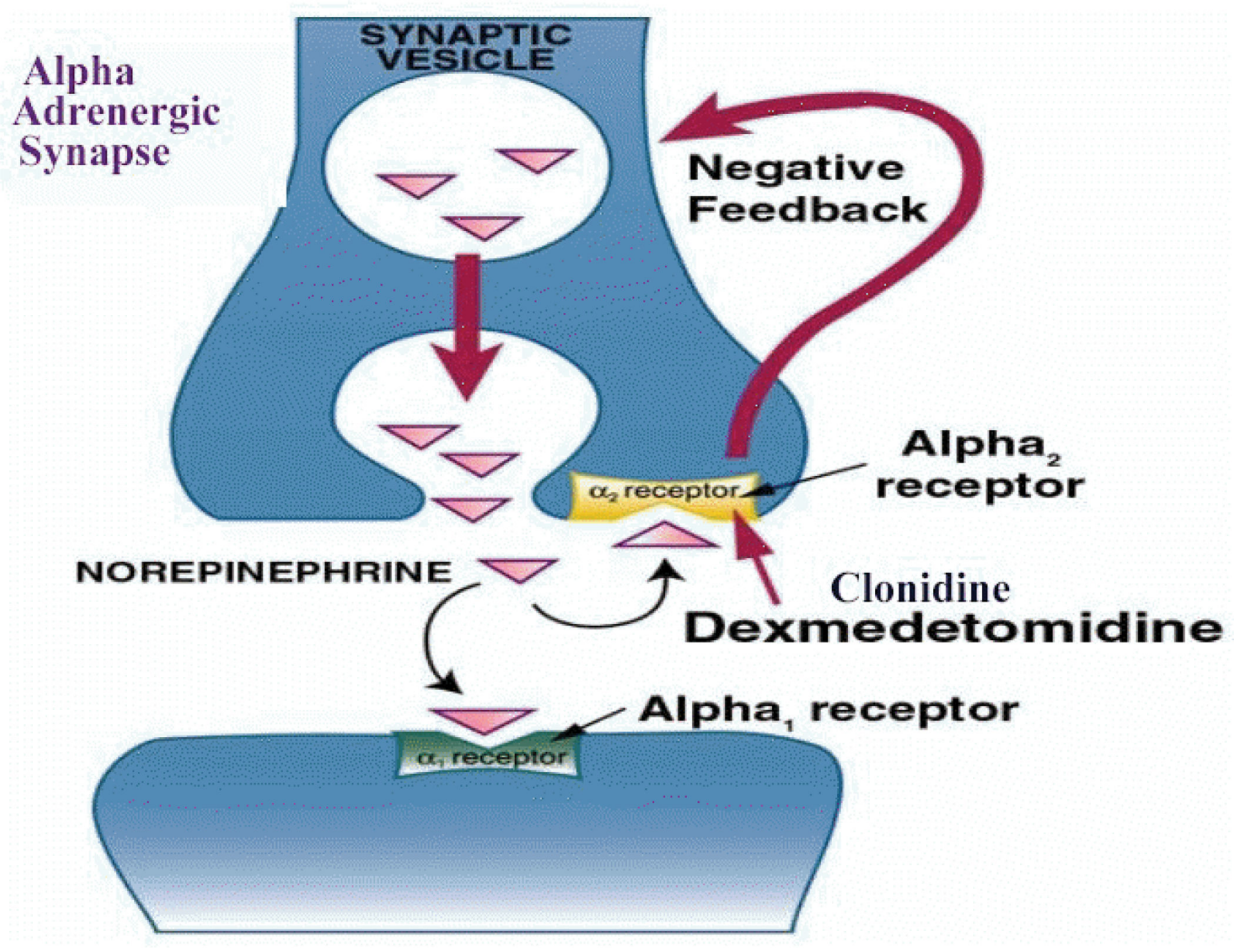
Schematic description of the mechanisms of dexmedetomidine which is much more selective for the presynaptic alpha 2 receptor than clonidine (adapted from dexmedetomidine.com).

Dexmedetomidine is an imidazole derivative that has been Food and Drug Administration approved since 1999. It is a highly selective alpha 2 agonist [1]. It is formulated as a clear colorless liquid in a concentration of 200 mcg/ml. It is eight times more selective for the presynaptic alpha 2 receptor than clonidine. It therefore provides better analgesia, more powerful sedation that the patient can be readily be aroused from and much less hypotension than clonidine [2]. Dexmedetomidine unlike clonidine is not an antihypertensive drug. When it is administered as a bolus to awake volunteers the blood pressure rises for five to ten minutes and then falls back to 10–20 percent below baseline [3,4]. Binding to the presynaptic alpha 2 receptor at the adrenergic synapse leads to negative feedback inhibition of norepinephrine release. It is this greater affinity for the presynaptic alpha 2 rather than the postsynaptic alpha 1 receptor that leads to the very different cardiovascular effects of dexmedetomidine and clonidine (Figure 1).

## Pharmacokinetics and Pharmacodynamics

Dexmedetomidine has a distribution half-life of six minutes and an elimination half-life of two hours. This long half-life makes it suitable for bolus administration even though in most studies it is administered by intravenous infusion. The volume of distribution is 118 liters and its clearance is 39 liters/hr. It is metabolized in the liver to inactive metabolites and it is 94% protein bound [3]. It acts at various sites in the brain. The most important is the locus coeruleus [5] which in English means the blue place. The blue color in unstained brain tissue is due to neuromelanin in the noradrenergic nerve cell bodies that are highly concentrated in this area [6]. The action in the locus coeruleus is thought to be the main mechanism of inducing sleep and sedation. Another important area of action is the substantia gelatinosa in the dorsal horn of the spinal cord that is thought to be a major site of analgesic action.

## Central Nervous System Actions

Dexmedetomidine produces co-operative sedation and anxiolysis. At higher doses sedation can be profound but the patient can be readily awakened from this sleeplike state. Dexmedetomidine induced sleep mimics natural sleep more closely than any other anesthesia drug and it is now used as a treatment for insomnia and sleep deprivation in critical care. It does not have strong amnestic properties like the benzodiazepines or propofol. It is therefore essential that some other drug with more powerful amnestic action be co-administered when things happen in the operating room that should better not be remembered by the patient. The clear headed anxiolysis without amnesia is highly desirable when the consent needs to be changed and when performing the pre-operative “huddle” in the operating room. Unlike with benzodiazepine sedation the patient on dexmedetomidine will not be disinhibited and will remember the change in surgical/anesthesia consent. Its sedation exhibits a powerful akinesia. Patients undergoing dexmedetomidine sedation move very little and this is highly desirable during sedation for computer tomography (CT) and magnetic resonance imaging (MRI) scans or when neuromuscular blocking drugs are contraindicated because of neuromonitoring requirements. Neuroexcitatory phenomena that are frequently associated with propofol and etomidate do not occur with dexmedetomidine.

Dexmedetomidine has powerful analgesic action without respiratory depression and reduces opioid requirements by more than fifty percent. It has been used successfully as a patient-controlled analgesia (PCA) drug in combination with opioids. This resulted in better analgesia and less nausea and vomiting than opioid alone [7]. There also appears to be a lower risk of developing addiction to alpha-2 agonists than to opioids and a more widespread intra-op and post-op use as part of PCA regimens could help stem the flow of the current opioid addiction epidemic in the USA.

Orientation in time and place and absence of post-operative delirium are the hallmark of demedetomidine sedation and it should form a major component of balanced anesthesia in all patients at risk of delirium [8,9]. There is significant evidence for a neuroprotective effect of dexmedetomidine following experimental cerebral ischemia [10]. It is a cerebral vasoconstrictor [11] and lowers intracranial pressure. This offers superior operating conditions for neurosurgeons in the operating room [12] and makes it an ideal sedative drug for neurocritical care in patients with raised ICP. It also lowers intraocular pressure which makes it a good choice for eye surgery and patients in steep Trendelenburg position [13].

## Cardiovascular System Actions

Bradycardia and transient hypertension are the main effects of bolus administration. The bradycardia is usually the dose limiting side effect. The transient hypertension is followed by a return of the blood pressure to 10–20 percent below baseline. The transient hypertension is probably due to alpha mediated direct peripheral vasoconstriction. Hypotension may occur when drugs with vasodilator action are co-administered because the ability of the sympathetic nervous system to compensate for this vasodilation will be impaired by dexmedetomidine. It is therefore essential to maintain a normal circulating blood volume and to reduce the amount of vasodilator drugs to prevent hypotension from occurring. If hypotension does occur, it will respond readily to phenylephrine or vasopressin. It may be advisable to start with a bolus dose of 0.25 micrograms/ kg and titrate to the heart rate and the level of sedation required. There is a marked inter-patient variability in dosage requirements with this drug and the recommended loading dose of 1 mcg/kg administered over 10 minutes is too much for some patients and insufficient for others.

There is reduced intraoperative heart rate variability and this has been associated with a reduction in myocardial infarction and stroke [14]. The negative chronotropic effect of dexmedetomidine is not associated with a negative inotropic effect and this makes it a good drug to control tachycardia in patients with congestive cardiac failure [15]. It is an effective drug to control tachycardia during intubation and has been found to be superior to fentanyl for this purpose [16]. The absence of respiratory depression also allows for more effective pre-oxygenation than when fentanyl is used for this purpose. This is especially important when the patient has to undergo a rapid sequence induction. Better ablation of airway reflexes with dexmedetomidine as compared to fentanyl also allows for easier mask ventilation with a lower incidence of laryngospasm prior to the onset of neuromuscular blockade. There have not been any reports of chest wall rigidity reported with dexmedetomidine. The opioid induced chest wall rigidity when larger doses of opioids are administered at the beginning of the case occasionally results in difficult mask ventilation when neuromuscular blockade has not yet been fully established. This problem does not occur with dexmedetomidine.

## Respiratory System Actions

Dexmedetomidine is not associated with significant respiratory depression and the respiratory rate does not slow down like it is the case with the use of opioids [17]. The end-tidal CO2 rise is minimal and oxygen saturations on pulse oximetry do not drop. This lack of respiratory depression is especially desirable in craniotomy and pulmonary hypertension patients post-operatively. Normal CO2 levels in the arterial blood in the recovery room will prevent exacerbation of raised intracranial pressure and pulmonary hypertension. Therefore, it is a much safer drug for these patients than opioids that are usually associated with hypercarbia and acidosis due to their respiratory depressant effects.

There is an excellent ablation of airway reflexes with dexmedetomidine [18,19]. This makes it an ideal agent for awake intubation, upper gastrointestinal endoscopy, ENT and vocal cord procedures and when the patient needs to remain intubated post-operatively. Better tolerance of the endotracheal tube with less coughing and gagging and shorter intubation times [20] may well lead to lower likelihood of having to progress to a tracheostomy in critical care patients. The incidence of laryngospasm is significantly reduced in high risk patients and it is an excellent induction agent when the patient needs to be intubated without a neuromuscular blocking agent.

Dexmedetomidine is a powerful bronchodilator and it reliably reverses histamine induced bronchospasm in experimental animals and it should be a first line agent for patients with asthma and chronic obstructive pulmonary disease [21].

## Gastrointestinal Actions

Dexmedetomidine is associated with much less impairment of gastrointestinal motility than the opioids and its use for ICU sedation prevents opioid induced ileus and enables patients to better absorb enteric feeding. There is no nausea and vomiting associated with it and it has been used successfully as a PCA drug in combination with opioids. The addition of dexmedetomidine to the opioid improved analgesia and reduced the incidence of nausea and vomiting [22].

## Renal Actions

Dexmedetomidine leads to an increase in urine output and has been demonstrated to have renal protective effects in experimental renal injury in rats [23]. It also was found to provide reno-protection against ischemia/reperfusion injury in mice [24]. It has also been demonstrated to lower the incidence of acute kidney injury (AKI) following cardiopulmonary bypass [25]. It was also associated with a reduced rate of AKI in another study of patients undergoing cardiac valve replacement [26].

## Endocrine Actions

Plasma catecholamine levels are significantly lower during dexmedetomidine-based anesthesia due to the negative feedback inhibition at adrenergic synapses [27]. This leads to better blood glucose control in the perioperative period [28]. The reduction in catecholamine levels prevents the increase in glycogenolysis and gluconeogenesis in the liver that is responsible for the stress induced rise in blood glucose [29].

## Clinical Uses of Dexmedetomidine

Dexmedetomidine has many clinical applications (Table 1). It is an excellent alternative to midazolam as a pre-operative anxiolytic. It prevents anxiety induced tachycardia and is associated with less hypotension than midazolam [30]. The clear headed nature of dexmedetomidine sedation allows the patient to be a mentally coherent member of the pre-operative huddle in the operating room. It can be used as an intravenous induction agent to prevent the tachycardia associated with intubation [16]. The transient hypertension during bolus administration can be controlled with simultaneous administration of propofol.

The profound ablation of airway reflexes reduces the risk of laryngospasm and the potent akinesia prevents the patient from moving under anesthesia which can be used in generalanesthesia with laryngeal mask airway. The near complete absence of respiratory depression of dexmedetomidine makes it the agent of choice in these patients for both anesthesia and post-operative analgesia for patients with obstructive sleep apnea and the obese. Studies show reduced post-operative desaturations [31] and re-intubation rates in sleep apnea patients may well be reduced. Dexmedetomidine also is a powerful bronchodilator and readily reverses histamine induced bronchospasm in experimental animals [21]. This makes it a first line agent for the treatment of bronchospasm induced by endotracheal intubation or the infusion of vancomycin. Because of these, it is an ideal agent for awake intubation and awake tracheostomy and is superior to fentanyl in this situation [32]. Dexmedetomidine also has a profound antisialogogue effect that is useful during fiber optic intubation. Dexmedetomidine is very useful in patients with asthma or COPD. It is the agent of choice for these patients because of its powerful bronchodilator action [21]. Prevention of bronchospasm eliminates problems like hypercarbia, air trapping and the dynamic hyperinflation syndrome. Dexmedetomidine is the best sedative drug when the patient needs to remain intubated post-operatively and neurologically evaluated on an ongoing basis. Excellent ablation of airway reflexes allows for better tolerance of the endotracheal tube. This allows for lower levels of sedation and a lower incidence of struggling against the ventilator. Endotracheal intubation associated tachycardia and hypertension, are also well controlled by this drug. However, dry mouth is a very common side effect of this drug (Table 2).

Motor evoked potential monitoring or nerve monitoring when there is a risk of surgically inducing damage to a nerve usually requires the absence of neuromuscular blocking agents. Most commonly the facial, sciatic and recurrent laryngeal nerves are monitored in this fashion. During spine surgery dexmedetomidine has been shown to not affect motor evoked potentials [33]. This is another useful property for patients under general anesthesia when neuromuscular blocking agents are not permitted.

Dexmedetomidine is an excellent agent for anesthesia for patients at risk of myocardial infarction or stroke. It is associated with reduced heart rate variability [34] and allows for excellent control of surgically induced sympathetic nervous system surges. This makes it less likely for atherosclerotic plaques to be lifted and prevents infarctions. Dexmedetomidine anesthesia has been associated with a lower rate of perioperative myocardial ischemia and stroke [14,34]. There is also a cerebral protective effect following experimental brain ischemia. This may well be due to an anti-inflammatory effect [35].

Dexmedetomidine is a valuable drug in the prevention of postoperative nausea and vomiting (PONV). It allows for a greater than 50% reduction in opioid requirements and this greatly reduces PONV. The addition of dexmedetomidine to an opioid in a patient-controlled anesthesia regimen has been shown to improve analgesia and reduce PONV and pruritus [36].

Dexmedetomidine is a very suitable agent for anesthesia for ear, nose and throat and vocal cord procedures. The excellent ablation of airway reflexes with dexmedetomidine provides good operating conditions during rigid laryngoscopy and reduces the requirement for neuromuscular blocking drugs. The blunting of the sympathetic nervous system response protects the patient from excessive tachycardia and hypertension. It is preferable to beta blockers for heart rate control during rigid laryngoscopy because it is a bronchodilator [21]. Beta blockers even when they are selective for the beta 1 receptor not infrequently precipitate bronchospasm in this patient population. There is a lower rate of laryngospasm in patients that are at high risk for it.

During thyroplasties when the patient needs to stay awake and co-operative there are fewer desaturations and apneas with dexmedetomidine than with opioid sedation. The patients remain fully co-operative and reliably respond to requests for phonation [37]. Another very important clinical use is during anesthesia for neurosurgery including awake craniotomy. Dexmedetomidine allows for early post-operative neurological evaluation of the patient even when the patient has to remain intubated. This reduces the need for follow up CT scans after completion of the surgery. There is less tachycardia and hypertension on emergence and this helps to reduce complications like myocardial ischemia and subdural hematoma. The absence of respiratory depression leads to near normal PaCO_2_ levels and prevents sedation induced exacerbation of intracranial pressure. During awake craniotomy the patients remain fully oriented in time, place and person. Dexmedetomidine also does not interfere with neuropsychological tests administered during the surgery. Unlike benzodiazepines, it does not cause any dysarthria. This is a great advantage because a very common reason for the patient to undergo a craniotomy awake is to monitor the speech function when the tumor is located in an eloquent part of the brain. When compared to propofol in a prospective randomized trial of awake craniotomy patients dexmedetomidine produced a shorter arousal time and a higher degree of surgeon satisfaction [38].

A very important clinical use of dexmedetomidine is during anesthesia for patients at risk of post-op delirium. It is associated with a significant reduction in the incidence of post-operative delirium [39,40]. Low dose nocturnal administration in the critical care unit has been show to prevent delirium in a randomized placebo-controlled trial [39]. When compared to propofol sedation it also has been shown to be associated with lower rates of delirium [40]. In children dexmedetomidine significantly reduced delirium and agitation in the PACU stage following tonsillectomy [41,42]. This effect is probably due to its opioid sparing properties because opioids are one of the main precipitants of post-operative confusion states.

Dexmedetomidine is a very useful agent during anesthesia for pediatric surgery including pediatric cardiac surgery. Children have a high incidence of postoperative agitation in the PACU. Dexmedetomidine is associated with a significant reduction in this incidence [41]. In pediatric cardiac surgery dexmedetomidine is very effective at preventing tachydysrhythmias [43,44]. Dexmedetomidine is beneficial in patients with systemic inflammatory response syndrome. An improved survival rate has been demonstrated with dexmedetomidine in endotoxin induced shock in rats [45] and it causes improved lactate clearance in critical care patients [46]. It has significant anti-inflammatory effects that may be responsible for this.

Dexmedetomidine is an ideal agent for monitored anesthesia care (MAC), because the patient remains co-operative and akinetic. It does not have any excitatory neurological effects like propofol and etomidate that occasionally precipitate unwanted myoclonic movements in the patient. The lack of patient movement during dexmedetomidine sedation makes it an ideal drug for CT or MRI scan sedation. It has been used extensively in pediatric procedural sedation outside the operating room [47]. When used during MAC it is essential to understand that dexmedetomidine does not have strong amnestic properties. At higher doses the patient will have the eyes closed but is likely to remember everything. It is essential to understand that the patient may be awake even though the eyes are closed. This is the opposite of ketamine anesthesia where the eyes remain open even when the patient is profoundly anesthetized. When things occur in the operating room that the patient should better not recall it is therefore essential to add another anesthesia drug with greater amnestic potency.

Dexmedetomidine should be used in patients at risk of peri-operative tachydysrhythmias. The majority of intra-operative tachydysrhythmias are caused by noxious surgical stimuli inducing an excess of catecholamine release. Dexmedetomidine effectively prevents the catecholamine release by negative feedback inhibition at the presynaptic membrane in adrenergic synapses. This leads to suppression of these tachydysrhythmias [43,44,48,49]. Dexmedetomidine is associated with lower heart rates and less intra-operative heart rate variability. It is also effective at suppressing tachydysrhythmias caused by sympathomimetic drugs like amphetamine and cocaine [50] and endocrine tumors like pheochromocytoma. Beta blockers are dangerous in these hyper catecholaminergic states as the beta blockade will remove the contractility from the left ventricle and leave the vasoconstrictive alpha effects of the catecholamines unopposed. This may result in a severe depression of cardiac output with cardiogenic shock and terminal lactic acidosis. Dexmedetomidine is a far safer method to lower heart rates in these patients than the use of beta blockers. It is useful in patients undergoing anesthesia for vascular surgery who have a high incidence of perioperative myocardial infarction and stroke. The central sympathectomy induced by dexmedetomidine helps to protect aneurysms from rupture and dissected blood vessels from extending their dissection during the application of noxious surgical stimuli. It has been used successfully to treat patients with cocaine induced aortic dissection [51].

Another clinical use of dexmedetomidine is in patients with renal impairment and kidney transplant patients. It has profound renal protective effects in experimental renal injury in rats [52]. It leads to an increase in urine output and it has been shown to lead to reduced AKI following cardiac surgery [25,53].

In postoperative management, dexmedetomidine has also been used successfully as a PCA drug in combination with opioids. The combination of dexmedetomidine with opioids had better analgesia, less nausea and vomiting, less pruritus and less gastrointestinal impairment than opioids alone [54]. It abolishes post-operative shivering [55]. It is therefore essential to keep the patient warm intraoperatively because the patient will not be able to raise his/her own body temperature via the shivering response in the recovery room. It is also effective to treat shivering in the recovery room in patients that did not receive dexmedetomidine intraoperatively. It has also been used as an adjunct to local anesthetics in spinal, epidural [56] and peripheral nerve block anesthesia to improve the quality and prolongs the duration of the analgesia.

Dexmedetomidine leads to improved blood sugar control. It is therefore a desirable drug to use in diabetics because the lower catecholamine levels lead to better blood sugar control. It suppresses the stress response to surgery and the concomitant rise in blood glucose better than fentanyl [57].

Nearly every patient will notice a dry mouth and this side effect can be beneficial during awake fiber optic intubation or rigid laryngoscopy. Bradycardia is usually the dose limiting side effect. If bradycardia occurs because too much dexmedetomidine has been administered too quickly it can be treated readily with atropine. Atropine is preferred to glycopyrrolate for serious bradycardia because it has stronger effect on cardiac muscarinic receptors [58]. Ephedrine and epinephrine can also be used to treat bradycardia if hypotension is present. Dexmedetomidine can be safely administered to patients on beta blockers as long as it is titrated carefully to the patient’s heart rate. There is no ceiling effect to the bradycardia seen with dexmedetomidine and excessive doses can precipitate an asystolic or bradycardic circulatory arrest (Table 2).

Transient hypertension nearly always occurs after bolus administration followed by a return of the blood pressure to 10–20 percent below baseline. Hypotension may become a problem when other drugs with vasodilator properties such as vancomycin are administered together with dexmedetomidine. This is because the dexmedetomidine induced sympathetic blockade impairs the body’s ability to compensate for the vasodilation with a rise in catecholamine levels. This situation can be readily corrected with the administration of vasopressors like phenylephrine or vasopressin. Slow wake up and excessive sedation in the recovery room have occasionally been noted with dexmedetomidine administration. This is preventable by limiting the administration of other sedative drugs like midazolam, opioids, propofol and inhalational anesthetics. In animals, there is a direct antagonist available named atipamezole. This drug is unfortunately not likely to ever become available in humans due to the high cost of getting a new drug like this licensed with the Food and Drug Administration. This is because reversal agents like flumazenil are not administered on a routine basis in clinical practice and the drug companies would be unlikely to ever recover their initial drug licensing costs. In certain situations dexmedetomidine should be avoided (Table 3).

## Summary

Dexmedetomidine is a drug that has many beneficial effects and should be used more frequently by anesthesia care providers to prevent common problems in the peri-operative period. There is however the caveat that a normal intravascular blood volume needs to be maintained at all times to prevent hypotension. Atropine should be readily available to treat bradycardia in case of inadvertent excessive administration. Bradycardic and asystolic cardiac arrests have been reported when excessive amounts of dexmedetomidine were administered. Judicious titration to effect will eliminate the occurrence of these events. It is very likely that this drug will ascend to take a much more prominent role in future anesthesia practice.

## Figures and Tables

**Table 1:** Clinical uses of dexmedetomidine.

Preoperative anxiolysis
Heart rate control during intubation
General anesthesia with laryngeal mask airway
When neuromuscular agents are not permitted
Anesthesia for patients at risk for myocardial infarction or stroke
Anesthesia for patients with sleep apnea and the obese
Anesthesia for patients with asthma and COPD
Prevention of postoperative nausea and vomiting (PONV)
Anesthesia for ear, nose and throat and vocal cord procedures
Awake intubation and awake tracheostomy
Anesthesia for neurosurgery including awake craniotomy
Anesthesia for patients at risk of postoperative delirium
Anesthesia for pediatric surgery including cardiac surgery
In patients with systemic inflammatory response syndrome (SIRS)
Monitored anesthesia Care (MAC)
Anesthesia for patients at risk of perioperative dysrhythmias
Anesthesia for vascular surgery
Anesthesia for patients with renal impairment and kidney transplants
When patients need to remain intubated postoperatively Patient controlled anesthesia
Postoperative shivering
Blood sugar control especially in diabetics
Adjunct to local anesthetics

**Note:** COPD: Chronic Obstructive Pulmonary Diseases.

**Table 2:** Side effects of dexmedetomidine.

Dry mouth
Bradycardia readily responds to atropine
Transient hypertension
Hypotension when administered together with vasodilator drugs
Slow wake up and excessive sedation in the recovery room

**Table 3:** Contraindications to dexmedetomidine.

Heart block
Severe bradycardia
Patients that are entirely dependent on an elevated sympathetic tone (hypovolemic, cardiogenic shock)
